# Bullying victimisation and perpetration and the association with mental disorders among adolescents in Kenya, Indonesia, and Vietnam: Findings from the National Adolescent Mental Health Surveys

**DOI:** 10.1186/s13034-025-00922-4

**Published:** 2025-07-31

**Authors:** Holly E. Erskine, Joemer C. Maravilla, Shoshanna L. Fine, Astha Ramaiya, Mengmeng Li, Amirah Ellyza Wahdi, Yohannes Dibaba Wado, Vu Manh Loi, Harvey A. Whiteford, David Lawrence, Hannah J. Thomas, James G. Scott

**Affiliations:** 1 School of Public Health, The University of Queensland, Herston, QLD Australia; 2 Queensland Centre for Mental Health Research, Wacol, QLD Australia; 3 Institute for Health Metrics and Evaluation, University of Washington, Seattle, WA USA; 4 Institute of Health Sciences and Nursing, Far Eastern University, Manila, Philippines; 5 Department of Mental Health, Johns Hopkins Bloomberg School of Public Health, Johns Hopkins University, Baltimore, MD USA; 6 Department of Population, Family and Reproductive Health, Johns Hopkins Bloomberg School of Public Health, Johns Hopkins University, Baltimore, MD USA; 7 Center for Reproductive Health, Faculty of Medicine, Public Health, and Nursing, Universitas Gadjah Mada, Yogyakarta, Indonesia; 8 Department of Biostatistics, Epidemiology, and Population Health, Faculty of Medicine, Public Health, and Nursing, Universitas Gadjah Mada, Yogyakarta, Indonesia; 9 African Population and Health Research Center, Nairobi, Kenya; 10 Institute of Sociology, Vietnam Academy of Social Sciences, Hanoi, Vietnam; 11 School of Population Health, Curtin University, Perth, WA Australia; 12 QIMR Berghofer Medical Research Institute, Brisbane, QLD Australia; 13 Child Health Research Centre, The University of Queensland, South Brisbane, QLD Australia; 14 Child and Youth Mental Health Service, Children’s Health Queensland, South Brisbane, QLD Australia

**Keywords:** Bullying, Victimisation, Perpetration, Mental disorders, Adolescent, Low- and middle-income countries, Kenya, Indonesia, Vietnam

## Abstract

**Background:**

Few studies report the prevalence of both bullying victimisation and perpetration at the national level in low- and middle-income countries, with fewer still reporting the association with mental disorders assessed diagnostically.

**Methods:**

Nationally representative household surveys of adolescents aged 10–17 years and their primary caregiver were conducted in Kenya, Indonesia, and Vietnam as part of the National Adolescent Mental Health Surveys (NAMHS). Adolescents were asked about bullying victimisation and perpetration in the past three months. The prevalence of mental disorders in the past 12 months was assessed using a diagnostic instrument. The prevalence of bullying involvement was calculated, along with bullying victimisation and perpetration which were further disaggregated by sex and age. Types of victimisation and perpetration were assessed among those who endorsed bullying involvement. Adjusted odds ratios quantified the association between any mental disorder and bullying victimisation and perpetration. All findings were weighted to the respective country’s population and presented with 95% confidence intervals (CIs).

**Results:**

The prevalence of any bullying involvement was highest in Kenya (6.3%, CI 5.3–7.4), followed by Indonesia (3.4%, 95% CI 2.4–4.8), then Vietnam (1.9%, 95% CI 1.3–2.7). For bullying victimisation, both Kenya (4.1%, 95% CI 3.4–5.0) and Indonesia (2.6%, 95% CI 1.9–3.6) had significantly higher prevalence than Vietnam (1.2%, 95% CI 0.9–1.67). Kenya had significantly higher prevalence of bullying perpetration (3.1%, 95% CI 2.5–3.8) compared to Indonesia (1.1%, 95% CI 0.6–2.1) and Vietnam (0.8%, 95% CI 0.5–1.2). Adolescents experiencing bullying victimisation were significantly more likely to have a mental disorder than those who had not been bullied in all three countries (Kenya: aOR 2.17, 95% CI 1.50–3.15; Indonesia: aOR 3.55, 95% CI 1.47–8.59; Vietnam: aOR 4.71, 95% CI 1.82–12.21). The same was seen for bullying perpetration although only in Kenya (aOR 4.38, 95% CI 2.77–6.93) and Indonesia (aOR 4.32, 95% CI 1.62–11.51).

**Conclusions:**

NAMHS is the first study to report national prevalence estimates of bullying victimisation and perpetration among adolescents in Kenya, Indonesia, and Vietnam. Bullying was strongly associated with adolescent mental disorders and is consequently an important consideration for strategies aimed at improving adolescent mental health.

**Supplementary Information:**

The online version contains supplementary material available at 10.1186/s13034-025-00922-4.

## Background

Bullying is a distinct form of aggression that is repeated, intended to cause harm, and occurs in the context of a power imbalance between the perpetrator and victim [1]. It is recognised as a health, education, and social justice issue on global progress agendas. For example, the Sustainable Development Goals (SDGs) have identified bullying victimisation (*being bullied)* as a specific indicator (4.a.2) under Goal 4 *‘Ensure inclusive and equitable quality education and promote lifelong learning opportunities for all’* [2–4]. Further, the Global Burden of Disease Study (GBD) now recognises bullying victimisation during childhood and adolescence as a risk factor for depressive disorders and anxiety disorders, quantifying the burden attributable to bullying victimisation for these two disorders globally and across 204 countries [5]. This is an important step forward in the recognition of bullying and its impact, given GBD estimates are used by governments and international organisations to inform policies, resource allocation, and funding priorities.

Bullying victimisation and perpetration can have significant impacts on a young person’s health, wellbeing, and functioning across multiple life domains. This includes an increased risk of poor mental health, suicidal behaviours, self-harm, substance use, poor educational outcomes, and reduced physical health, with the impacts occurring both during the formative years and well into adulthood [6–13]. While experiences of bullying can involve different behaviours (e.g., physical, verbal, relational, cyber), evidence indicates that all forms of bullying are associated with harm [14, 15].

The demographic profile of bullying is complex, with some studies finding that males are more likely to be bullied and bully others, although other studies suggest that sex differences may be less prominent in adolescence once specific types of bullying behaviours are accounted for, i.e., as males and females may be differentially involved in specific types of bullying (i.e., physical, verbal, relational, and cyber) [16–18]. Evidence also suggests that those who are both victimised and bully others are at greater risk of adverse mental health outcomes than those who report only victimisation or perpetration, noting that all three mutually exclusive categories of bullying involvement (victimisation-perpetration, victimisation only, perpetration only) have distinct profiles of adverse mental health outcomes [19, 20]. The relationship between bullying and poor mental health is further complicated by longitudinal studies demonstrating the bidirectionality of the relationship, i.e., whereby those with existing mental health problems are more likely to be bullied or bully others [21, 22].

Several studies have reported the prevalence of bullying victimisation among adolescents across both high-income countries (HICs) and low- and middle-income countries (LMICs) [23–28], mostly using data from cross-national studies such as the Global School-based Student Health Survey (GSHS) [29, 30] and the Health Behaviour in School-Aged Children survey (HBSC) [31]. One study utilising GSHS data for adolescents aged 12–17 years from 83 countries (inclusive of HICs and LMICs) found a pooled global prevalence of 30.5% for bullying victimisation on one or more days in the past 30 days, with country-specific prevalence ranging from 7 to 75% [26]. A study utilising data from the HBSC (inclusive of 40 LMICs and HICs from Europe and North America) found 16.2% of adolescents aged 11–15 years reported having experienced bullying victimisation 2 or 3 times a month or more in the past 2 months [27].

The same study of data from the HBSC further reported bullying involvement for victimisation only (12.6%), perpetration only (10.7%), and victim-perpetration (3.6%) [27]. In comparison, a nationally representative study assessing the 12-month prevalence of bullying among Australian adolescents aged 11–17 years found 13.3% reported victimisation only, although this study found lower prevalence of those who reported perpetration only (1.6%) and those who reported both (1.9%) [32]. Compared to the number of studies reporting the prevalence of bullying victimisation, far fewer studies measure bullying perpetration. As a result, it is often not possible to disaggregate those who are solely victimised from those who are both victimised and perpetrate bullying.

While, collectively, prevalence data for bullying in adolescents is available for several countries, studies differ in their approach to operationalising the definition of bullying. The widely accepted conceptual definition regards bullying as repeated actions intended to hurt or harm and, that over time, produce a power imbalance favouring the perpetrator [33]. The degree to which this definition is operationalised in measurement tools continues to vary widely, further complicated by differences in recall periods ranging from the past 30 days to the past 12 months [34, 35]. Victimisation or perpetration which occurs ‘occasionally’ or ‘once or twice’, is considered by many studies to satisfy the requirement of repetition, while others argue that this does not provide sufficient distinction between peer victimisation/aggression and bullying victimisation/perpetration [32, 36, 37]. The variation in what is considered to meet the threshold of bullying has further implications for studies investigating the association with mental disorders, whereby the strength of the association potentially becomes weaker or more obscured when the requirement of repetition is relaxed.

Parallel challenges exist in relation to how mental health-related outcomes are measured when investigating their association with bullying. Most studies of bullying use symptom measures to screen for mental health problems, rather than diagnostic instruments designed to elicit mental disorder diagnoses. While these symptom scales provide an indication of population-level psychopathology, they are unable to generate estimates for mental disorders defined consistently according to international classifications. As bullying is a modifiable risk factor for mental disorders [38], understanding the epidemiology of bullying and the association with mental disorders is critical to informing interventions which might reduce mental disorders in adolescents. While there is a need for efficient and evidence-based interventions in all countries, this is even more imperative for LMICs which have large proportions of adolescents in their population yet more limited resources to distribute among competing health interests.

The National Adolescent Mental Health Surveys (NAMHS) are representative household surveys of mental disorders among adolescents aged 10–17 years in Kenya, Indonesia, and Vietnam [39]. As part of the survey instrument in all three countries, questions about bullying victimisation and perpetration were included alongside diagnostic measures of mental disorders. This provided a unique opportunity to report the prevalence of bullying in adolescents living in three LMICs and examine the association with mental disorders. The current study utilises data from NAMHS to first report the overall prevalence of any bullying involvement among adolescents in Kenya, Indonesia, and Vietnam, further disaggregated into the three mutually exclusive categories of victimisation only, victimisation-perpetration, and perpetration only. The prevalence of overall bullying victimisation and perpetration, respectively, is then reported and further disaggregated by age and sex. The profile of bullying victimisation and perpetration is also assessed by calculating the proportions of specific types of victimisation and perpetration among those who have been bullied or bully others. Finally, this study reports the association between bullying victimisation and perpetration and mental disorders.

## Methods

### Sample

NAMHS involved nationally representative household surveys of mental disorders among adolescents aged 10–17 years and their primary caregiver in Kenya, Indonesia, and Vietnam [39, 40]. Households were randomly selected as per the respective survey’s sampling frame [39], and a reference adolescent was randomly selected if more than one adolescent in a household was in scope for the survey. Consent and assent were sought from the primary caregiver and adolescent, respectively, by a trained interviewer. The interviewer administered the instrument to the primary caregiver and adolescent, separately and privately, using a tablet or smartphone. Data collection occurred in 2021, with final samples consisting of 5,155 (Kenya), 5,664 (Indonesia), and 5,996 (Vietnam) households with complete data (i.e., complete primary caregiver and adolescent pairs). This equated to response rates of 97.9% (Kenya), 92.2% (Indonesia), and 81.1% (Vietnam). Full details of NAMHS, including the methodology, have been published elsewhere [39, 40].

### Measures

The measures included in NAMHS were developed collaboratively between the lead NAMHS organisations from five countries including Australia, Kenya, Indonesia, Vietnam, and the US. The measures utilised in NAMHS included diagnostic assessment of mental disorders, a range of risk and protective factors (including bullying), service use, and other information including demographics and COVID-19 impact. All measures were translated into the respective language of the country and underwent back-translation to English, review by in-country clinicians, adjustments after the pilot study phase, and revisions over an 18-month period prior to data collection [39, 40]. This was done collaboratively between all teams to ensure consistency across all three countries. Comprehensive descriptions of the measures, including their development and adaptation, have been published elsewhere [39, 40]. The measures described below are those pertinent to this study.

#### Bullying

Bullying victimisation and perpetration in the past three months were the primary outcome measures of the current study. Adolescents were asked about both bullying victimisation and perpetration, with these questions partly adapted from the GSHS [29] and the Bullying and Cyberbullying Scale for Adolescents (BCS-A) [41]. Prior to answering the bullying questions, adolescents were read the following definition taken from the BCS-A [41] which covered the three defining principles of bullying– intention to harm, repetition, and power imbalance:

“Bullying is when young people intentionally hurt other young people by teasing, threatening, spreading rumours, excluding, or physically harming them. It is bullying when it happens over and over again and it is difficult for the young person to make it stop. This can happen in person or online. It is not bullying when it is done in a friendly way or when two people of the same strength or power argue or fight.”

Bullying victimisation was assessed by the question “In the past 3 months, how often have you been bullied?” with response options of “Never”, “Once or twice”, “Every few weeks”, “About once a week”, “A few days a week”, and “Most days”. Bullying perpetration was assessed by an equivalent question: “In the past 3 months, how often have you bullied another young person?” with the same response options. Those who endorsed the overall or ‘global’ question were then asked about specific types (i.e., physical verbal, relational, and cyber) of victimisation and perpetration. These questions are shown in Table 1.

**Table 1:** Questions about specific types of victimisation and perpetration

Type	Victimisation	Perpetration
Physical	Been hit, kicked, pushed, shoved around, or locked indoors?	Hit, kicked, or pushed another young person, shoved them around, or locked them indoors?
Verbal	Been called mean names, made fun of, or teased in a hurtful way?	Called another young person mean names, made fun of them, or teased them in a hurtful way?
Relational	Been left out of things on purpose, excluded from a group of friends, or completely ignored?	Left another young person out of things on purpose, excluded them from your group of friends, or completely ignored them?
	Had lies told or false rumours spread about you to try and make others dislike you?	Told lies or spread false rumours about another young person to try and get others to dislike them?
Cyber	Been cyberbullied? That involves being bullied through email, social media, gaming, video sharing, or instant messaging.	Cyberbullied another young person? That involves bullying through email, social media, gaming, video sharing, or instant messaging.

Bullying victimisation and perpetration were each defined as those who had endorsed a frequency of “Every few weeks”, “About once a week”, “A few days a week”, or “Most days” for the respective question. Those who responded “Never” or “Once or twice” were classified as not having endorsed bullying. This threshold was applied in line with bullying literature to reflect the principle of repetition in the established definition of bullying and to clearly delineate bullying from the broader experience of peer victimisation/aggression [34, 36, 42, 43]. Those who gave a non-meaningful response (“Don’t know” or “Prefer not to say”) were classified as missing and omitted from the relevant analysis, with the numbers of missing data reported in the footnote of the relevant table for each analysis. Weighted proportions and unweighted numbers of adolescents endorsing each response option for bullying victimisation and perpetration, respectively, are available in Table S1 in Additional File 1.

#### Mental disorders

Mental disorders were grouped into a single category of any mental disorder (of the six mental disorders in scope) in the past 12 months. Mental disorders were measured using the Diagnostic Interview Schedule for Children, Version 5 (DISC-5) [44, 45], a structured diagnostic interview which provides diagnoses of mental disorders according to the Diagnostic and Statistical Manual of Mental Disorders, Fifth Edition (DSM-5) [46]. The DISC-5 is designed to be administered by trained lay interviewers, i.e., those with no formal clinical training but who are trained to administer the DISC-5. The six mental disorders included in NAMHS were social phobia, generalised anxiety disorder, major depressive disorder, conduct disorder, posttraumatic stress disorder (PTSD), and attention-deficit/hyperactivity disorder (ADHD), which were chosen due to their prevalence and burden of disease among adolescents [39]. Diagnostic modules for all mental disorders were administered to the adolescent except for ADHD, which was administered to the primary caregiver. The DISC-5 assesses prevalence of each disorder in scope in the past 12 months, following diagnostic algorithms which require endorsement of relevant symptoms and a level of impairment as per DSM-5 diagnostic criteria [46].

#### Demographics

Demographic characteristics were used as adjustments in relevant logistic regression models. Demographic information was reported by the primary caregiver, including the sex, age, and current school attendance of the adolescent. Items from the wealth index [47] were also administered to the primary caregiver to generate an indicator of household wealth. Household wealth quintiles were calculated utilising methodology adapted from the Demographic and Health Surveys (DHS) [47] and standardised across all analyses of NAMHS data [39]. Urbanicity (urban and rural) was determined by the household’s location and was incorporated into the sampling frame of each survey to allow for disaggregation at this level [39].

### Analysis

The prevalence of any bullying involvement (i.e., victimisation or perpetration) was generated for Kenya, Indonesia, and Vietnam, respectively. Bullying involvement was further disaggregated into three mutually exclusive categories: victimisation only, victimisation-perpetration, perpetration only. The overall prevalence of bullying victimisation (regardless of perpetration) and perpetration (regardless of victimisation) was generated, and further disaggregated by sex (male and female) and age group (10–14 years and 15–17 years). These age groups were chosen given their consistency with other studies utilising 5-year age groups across the lifespan, e.g., GBD [48], the definition of ‘early adolescence’ as operationalised by other studies (e.g., the Global Early Adolescent Study) [49], and the approach used to report key NAMHS findings in previous publications [39]. In addition, among those who endorsed bullying victimisation and perpetration, the proportion reporting any frequency of physical, verbal, relational, and cyber victimisation and perpetration was calculated.

The prevalence of any mental disorder in the past 12 months was compared between those who had experienced bullying victimisation and those who had not. The same comparison was made for bullying perpetration. The association between bullying victimisation and perpetration with any mental disorder in the past 12 months was quantified using logistic regression. Multivariable analyses adjusted the models for demographic variables including adolescent age, adolescent sex, adolescent current school attendance, household wealth, and urbanicity. Given bullying victimisation and perpetration were assessed in the same model, the model further controlled for the effect of the other type of bullying involvement (i.e., the adjusted odds ratios [aORs] for bullying victimisation accounted for the impact of perpetration, and vice versa).

All analyses were weighted utilising the relevant population weight for each country [39] and presented with 95% confidence intervals (CIs). All analyses were conducted in Stata17 [50].

## Results

The overall prevalence of any bullying involvement (i.e., victimisation or perpetration) was highest in Kenya (6.3%, 5.3–7.4%), followed by Indonesia (3.4%, 95% CI 2.4–4.8) then Vietnam (1.9%, 95% CI 1.3–2.7) (Table 2). When disaggregating further into the three mutually exclusive categories of bullying involvement, the difference in prevalence between Kenya and Indonesia was no longer significant for victimisation only (Kenya: 3.2%, 95% CI 2.5–3.9; Indonesia: 2.3%, 95% CI 1.7–3.1); although Vietnam remained significantly lower (1.1%, 95% CI 0.8–1.6). For victimisation-perpetration, the only significant difference was seen between Kenya (1.0%, 95% CI 0.7–1.3) and Vietnam (0.1%, 95% CI <0.1–0.3), with Indonesia not differing significantly from either country (0.3%, 95% CI 0.1–0.8). The prevalence of perpetration only was significantly higher in Kenya (2.1%, 95% CI 1.7–2.7) as compared to Indonesia (0.8%, 95% CI 0.4–1.5) and Vietnam (0.7%, 95% CI 0.4–1.1).

**Table 2:** Bullying involvement in the past three months among adolescents in Kenya, Indonesia, and Vietnam

	Kenya		Indonesia		Vietnam	
	% (95% CI)	*n*	% (95% CI)	*n*	% (95% CI)	*n*
Bullying victimisation or perpetration	6.3 (5.3–7.4)	333	3.4 (2.4–4.8)	167	1.9 (1.3–2.7)	121
Victimisation only	3.2 (2.5–3.9)	165	2.3 (1.7–3.1)	102	1.1 (0.8–1.6)	66
Victimisation-perpetration	1.0 (0.7–1.3)	52	0.3 (0.1–0.8)	18	0.1 (< 0.1–0.3)	7
Perpetration only	2.1 (1.7–2.7)	116	0.8 (0.4–1.5)	47	0.7 (0.4–1.1)	48

Proportions (%) and 95% CIs are weighted. Numbers (n) are unweighted

Total sample after omitting ‘Don’t know’ and ‘Prefer not to say’ to both the victimisation AND perpetration questions: Kenya = 5147; Indonesia = 5584; Vietnam = 5835

**Fig. 1:**
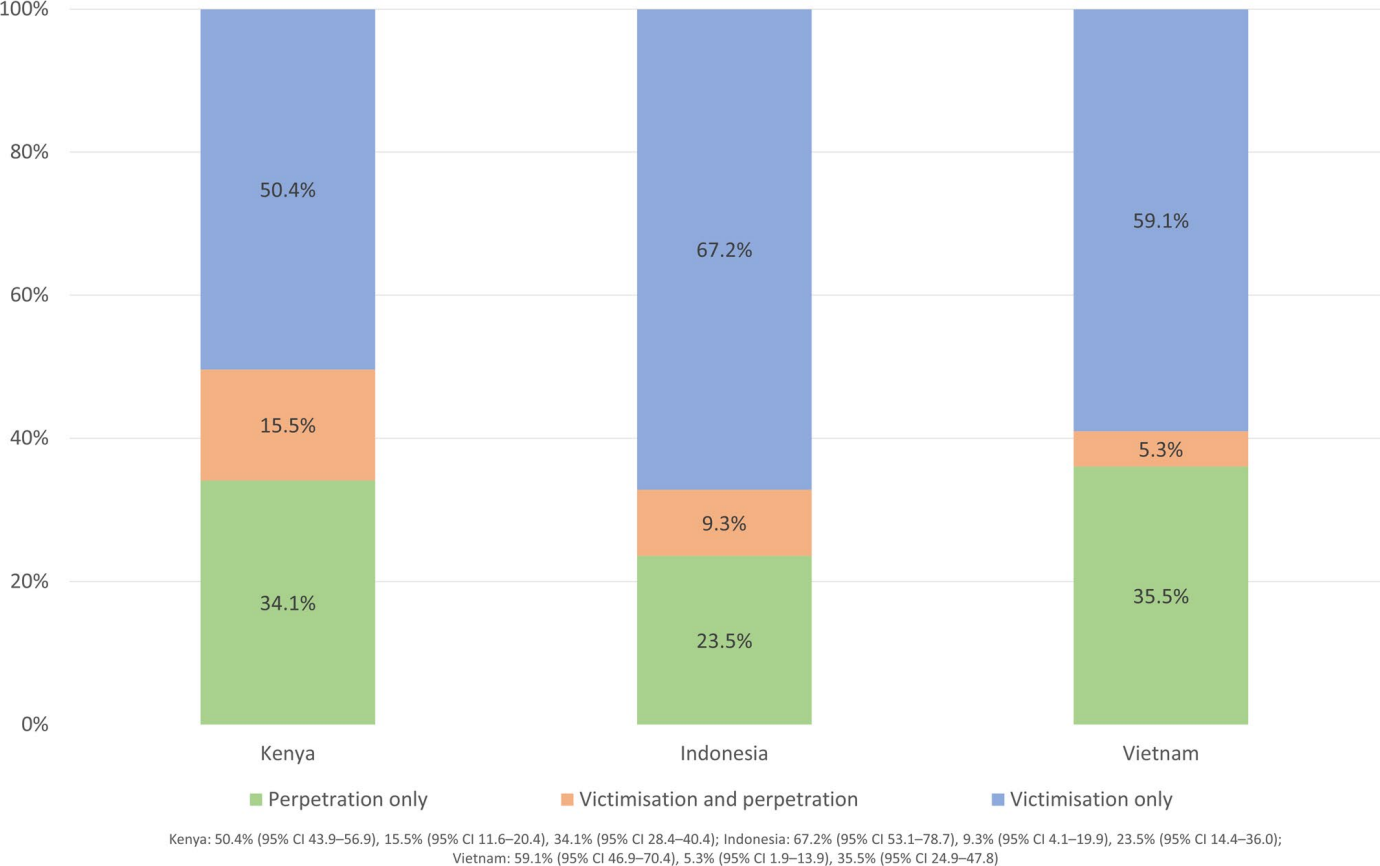
Distribution of bullying involvement in the past three months among adolescents in Kenya, Indonesia, and Vietnam

Figure 1 shows the proportion of each category of bullying involvement among adolescents who had any involvement in bullying in Kenya (6.3%, *n* = 333), Indonesia (3.4%, *n* = 167), and Vietnam (1.9%, *n* = 121). No significant differences were found between countries.

As shown in Table 3, the prevalence of bullying victimisation (regardless of perpetration) differed significantly by country. Kenya had the highest prevalence (4.1%, 95% CI 3.4–5.0), followed by Indonesia (2.6%, 95% CI 1.9–3.6) then Vietnam (1.2%, 95% CI 0.9–1.7). There was no difference in the prevalence of bullying victimisation by sex in any country. However, younger adolescents aged 10–14 years demonstrated higher prevalence of bullying victimisation than older adolescents aged 15–17 years in both Kenya (10–14 years: 4.9%, 95% CI 4.0–5.9; 15–17 years: 2.6%, 95% CI 1.9–3.6) and Indonesia (10–14 years: 3.4%, 95% CI 2.4–4.8; 15–17 years: 1.1%, 95% CI 0.6–2.1). No significant difference by age was seen in Vietnam.

Bullying perpetration (regardless of victimisation) was also significantly higher in Kenya (3.1%, 95% CI 2.5–3.8) as compared to Indonesia (1.1%, 95% CI 0.6–2.1) and Vietnam (0.8%, 95% CI 0.5–1.2), although no significant difference was seen between the latter two countries (Table 3). Bullying perpetration demonstrated a somewhat different demographic profile to bullying victimisation in all three countries. No difference in bullying perpetration was seen by sex or age in any country.

**Table 3:** Bullying victimisation and perpetration in the past three months among adolescents in Kenya, Indonesia, and Vietnam

	Kenya		Indonesia		Vietnam	
	% (95% CI)	*n*	% (95% CI)	*n*	% (95% CI)	*n*
**Bullying victimisation**	4.1 (3.4–5.0)	217	2.6 (1.9–3.6)	120	1.2 (0.9–1.7)	74
Sex						
Male	4.6 (3.6–5.8)	118	2.9 (1.9–4.3)	66	1.3 (0.9–2.0)	42
Female	3.7 (2.9–4.6)	99	2.3 (1.6–3.3)	54	1.1 (0.7–1.7)	32
Age						
10–14 years	4.9 (4.0–5.9)	167	3.4 (2.4–4.8)	91	1.4 (1.0–2.1)	51
15–17 years	2.6 (1.9–3.6)	50	1.1 (0.6–2.1)	29	0.8 (0.4–1.5)	23
**Bullying perpetration** ^b^	3.1 (2.5–3.8)	168	1.1 (0.6–2.1)	66	0.8 (0.5–1.2)	56
Sex						
Male	3.4 (2.7–4.3)	91	1.6 (0.9–2.8)	51	1.2 (0.7–1.9)	47
Female	2.8 (2.1–3.7)	77	0.6 (0.2–1.5)	15	0.3 (0.1–0.8)	9
Age						
10–14 years	3.2 (2.6–4.0)	114	0.9 (0.4–1.8)	30	0.7 (0.4–1.2)	33
15–17 years	2.9 (2.2–4.0)	54	1.6 (0.8–3.1)	36	0.9 (0.5–1.7)	23

Proportions (%) and 95% CIs are weighted. Numbers (n) are unweighted

^a^Bullying victimisation inclusive of those who reported victimisation only and those who reported victimisation-perpetration

^b^Bullying perpetration inclusive of those who reported perpetration only and those who reported victimisation-perpetration

Total sample for bullying victimisation after omitting ‘Don’t know’ and ‘Prefer not to say’: Kenya = 5152; Indonesia = 5610; Vietnam = 5883

Total sample for bullying perpetration after omitting ‘Don’t know’ and ‘Prefer not to say’: Kenya = 5149; Indonesia = 5624; Vietnam = 5925

The proportions of the four different types of victimisation among adolescents who endorsed bullying victimisation are shown in Table 4. Among adolescents who had been bullied (Kenya = 217; Indonesia = 120; Vietnam = 74), the proportion reporting verbal (Kenya: 79.1%, 95% CI 72.2–84.7; Indonesia: 81.6%, 95% CI 69.9–89.5; Vietnam: 71.3%, 95% CI 53.6–84.2) and relational (Kenya: 60.6%, 95% CI 51.3–69.1; Indonesia: 66.1%, 95% CI 53.5–76.8; Vietnam: 58.5%, 95% CI 42.8–72.6) victimisation was similar across all three countries. However, the proportion of these adolescents who reported physical victimisation was significantly lower in Indonesia (22.2%, 95% CI 14.8–31.8) as compared to Kenya (58.6%, 95% CI 50.8–66.0) and Vietnam (55.5%, 95% CI 40.2–69.8). The proportion of cyber victimisation among adolescents who had been bullied was low across all three countries (Kenya: 4.7%, 95% CI 2.5–8.7; Indonesia: 11.4%, 6.8–18.6; Vietnam: 15.8%, 95% CI 8.0–28.7). Among those reporting bullying perpetration (Kenya = 168; Indonesia = 66; Vietnam = 56), the profile of bullying was largely consistent with victimisation. However, no significant difference for physical perpetration was seen between Indonesia (47.5%, 95% CI 31.5–64.0), Kenya (66.7%, 95% CI 57.8–74.6), and Vietnam (72.8%, 95% CI 50.2–87.7).

**Table 4:** Proportion of each type of victimisation/perpetration among adolescents reporting bullying victimisation and perpetration, respectively, in the past three months in Kenya, Indonesia, and Vietnam

	Kenya		Indonesia		Vietnam	
	% (95% CI)	*n*	% (95% CI)	*n*	% (95% CI)	*n*
**Victimisation**						
Physical	58.6 (50.8–66.0)	123	22.2 (14.8–31.8)	31	55.5 (40.2–69.8)	44
Verbal	79.1 (72.2–84.7)	170	81.6 (69.9–89.5)	97	71.3 (53.6–84.2)	49
Relational	60.6 (51.3–69.1)	138	66.1 (53.5–76.8)	80	58.5 (42.8–72.6)	44
Cyber	4.7 (2.5–8.7)	11	11.4 (6.8–18.6)	24	15.8 (8.0–28.7)	12
**Perpetration**						
Physical	66.7 (57.8–74.6)	108	47.5 (31.5–64.0)	25	72.8 (50.2–87.7)	37
Verbal	75.1 (66.3–82.2)	122	85.1 (71.4–92.9)	52	64.7 (46.6–79.3)	37
Relational	57.6 (48.6–66.1)	95	51.2 (32.9–69.3)	33	42.4 (28.4–57.8)	25
Cyber	7.7 (4.0–14.1)	13	37.5 (15.9–65.5)	23	12.8 (6.5–23.7)	6

Proportions (%) and 95% CIs are weighted. Numbers (n) are unweighted

Samples among bullying victimisation after omitting ‘Don’t’ know’ and ‘Prefer not to say’: Physical: Kenya = 217; Indonesia = 120; Vietnam = 72; Verbal: Kenya = 217; Indonesia = 118; Vietnam = 72; Relational: Kenya = 217; Indonesia = 119; Vietnam = 72; Cyber: Kenya = 216; Indonesia = 119; Vietnam = 70

Samples among bullying perpetration after omitting ‘Don’t’ know’ and ‘Prefer not to say’: Physical: Kenya = 168; Indonesia = 66; Vietnam = 55; Verbal: Kenya = 168; Indonesia = 66; Vietnam = 55; Relational: Kenya = 168; Indonesia = 66; Vietnam = 56; Cyber: Kenya = 168; Indonesia = 66; Vietnam = 56

Table 5 shows the prevalence of any mental disorder in the past 12 months among adolescents who had experienced bullying victimisation and perpetration in the past 3 months compared to those who had not. In all three countries, those who had experienced bullying victimisation (Kenya: 28.2%, 95% CI 22.2–35.2; Indonesia: 19.7%, 95% CI 10.5–34.0; Vietnam: 13.3%, 95% CI 5.2–29.9) had significantly higher prevalence of mental disorders than those who had not experienced bullying victimisation (Kenya: 11.5%, 95% CI 10.3–12.8; Indonesia: 5.0%, 95% CI 4.0–6.2; Vietnam: 3.1%, 95% CI 2.5–3.9). In Kenya and Indonesia, the prevalence of any mental disorder in the past 12 months was significantly higher among adolescents who reported bullying perpetration (Kenya: 40.7%; 95% CI 31.7–50.4; Indonesia: 27.4%, 95% CI 14.5–45.6) compared to those who did not (Kenya: 11.2%; 95% CI 10.0–12.5; Indonesia: 5.2%, 95% CI 4.1–6.6) (Table 5). No significant difference by bullying perpetration was seen in Vietnam.

**Table 5:** Prevalence of any mental disorder in the past 12 months by bullying victimisation and perpetration in the past three months among adolescents in Kenya, Indonesia, and Vietnam

	Any mental disorder in the past 12 months					
	Kenya		Indonesia		Vietnam	
	% (95% CI)	*n*	% (95% CI)	*n*	% (95% CI)	*n*
**Victimisation** ^a^						
Yes	28.2 (22.2–35.2)	56	19.7 (10.5–34.0)	22	13.3 (5.2–29.9)	11
No	11.5 (10.3–12.8)	576	5.0 (4.0–6.2)	289	3.1 (2.5–3.9)	171
**Perpetration** ^b^						
Yes	40.7 (31.7–50.4)	64	27.4 (14.5–45.6)	14	5.8 (1.9–16.2)	5
No	11.2 (10.0–12.5)	567	5.2 (4.1–6.6)	303	3.2 (2.6–4.0)	177

Proportions (%) and 95% CIs are weighted. Numbers (n) are unweighted

^a^Bullying victimisation inclusive of those who experienced victimisation only and those who experienced both victimisation and perpetration

^b^Bullying perpetration inclusive of those who experienced perpetration only and those who experienced victimisation-perpetration

Table 6 presents the unadjusted ORs and aORs (adjusted for adolescent age, sex, and current school attendance as well as household wealth and urbanicity) for any mental disorder in the past 12 months among those who endorsed bullying victimisation and perpetration, respectively. After adjustment, adolescents in all three countries who experienced bullying victimisation were significantly more likely to meet criteria for any mental disorder in the past 12 months than those who had not experienced bullying victimisation (Kenya: aOR 2.17, 95% CI 1.50–3.18; Indonesia: aOR 3.55, 95% CI 1.47–8.59; Vietnam: aOR 4.71, 95% CI 1.82–12.21). Further, adolescents in Kenya (aOR 4.38, 95% CI 2.77–6.93) and Indonesia (aOR 4.32, 95% CI 1.62–11.51) who reported bullying perpetration were significantly more likely to have any mental disorder in the past 12 months as compared to those who did not bully others.

**Table 6:** Unadjusted and adjusted odds ratios for any mental disorder in the past 12 months by bullying victimisation and perpetration in the past three months among adolescents in Kenya, Indonesia, and Vietnam

	Kenya		Indonesia		Vietnam	
	Unadjusted OR (95% CI)	Adjusted OR (95% CI)^a^	Unadjusted OR (95% CI)	Adjusted OR (95% CI)^a^	Unadjusted OR (95% CI)	Adjusted OR (95% CI)^a^
**Victimisation** Reference: No victimisation	3.04 (2.18–4.24)	2.17 (1.50–3.15)	4.69 (2.29–9.62)	3.55 (1.47–8.59)	4.71 (1.85–11.96)	4.71 (1.82–12.21)
**Perpetration** Reference: No perpetration	5.44 (3.63–8.16)	4.38 (2.77–6.93)	6.88 (3.16–14.96)	4.32 (1.62–11.51)	1.85 (0.59–5.84)	1.41 (0.40–4.93)

^a^Adjusted for adolescent age, adolescent sex, adolescent current school attendance, household wealth, and urbanicity

## Discussion

NAMHS measured both bullying victimisation and perpetration in nationally representative surveys in Kenya, Indonesia, and Vietnam, further allowing investigation of the respective association with mental disorder at a national level. The prevalence of any bullying involvement (victimisation or perpetration) differed significantly between Kenya (6.3%), Indonesia (3.4%), and Vietnam (1.9%). Significant differences in prevalence were also seen for overall victimisation (Kenya: 4.1% and Indonesia: 2.6% vs. Vietnam: 1.2%) and perpetration (Kenya: 3.1% vs. Indonesia: 1.1% and Vietnam: 0.8%) although only between certain countries. Further, all three countries demonstrated significantly higher prevalence of any mental disorder in the past 12 months among those who had been bullied as compared to those who had not (Kenya: 28.2% vs. 11.5%; Indonesia: 19.7% vs. 5.0%; Vietnam: 13.3% vs. 3.1%). Both Kenya and Indonesia demonstrated significantly higher prevalence of any mental disorder among those who had bullied others compared to those who had not (Kenya: 40.7% vs. 11.2%; Indonesia: 27.4% vs. 5.2%).

Broadly, the prevalence of bullying victimisation found for Kenya, Indonesia, and Vietnam in NAMHS was lower than that of the large school-based studies available for these countries. While the operationalisation of repetition applied in this study may partially explain the low prevalence of bullying victimisation reported for NAMHS, this prevalence is still comparatively lower than estimates from other data applying a similar threshold. For example, the Kenya GSHS 2003 found approximately a quarter of 13–15-year-olds had experienced bullying victimisation (3–5 days or more) in the past 30 days [51]. Using the same threshold, the Indonesia GSHS 2015 and Vietnam GSHS 2013 found bullying victimisation prevalence of approximately 7% and 8%, respectively, among 13–17-year-olds [52, 53].

Multiple contextual and methodological factors may have contributed to the lower prevalence of bullying victimisation seen in NAMHS. For example, the majority of the NAMHS instrument, including the bullying questions, were administered by an interviewer. It is possible that adolescents may have been less inclined to disclose victimisation verbally to an adult as compared to written self-administered questionnaires [54, 55] which are used by many studies of bullying victimisation, including the GSHS and HBSC. However, compared to bullying victimisation, an equivalent magnitude difference is not seen for bullying perpetration when the findings of NAMHS (Kenya: 3.1% vs. Indonesia: 1.1% and Vietnam: 0.8%) are compared to other, albeit few, large-scale surveys, e.g., US: 6.6% [56], Australia: 3.5% [32], Jordan: 9.3% [57]. This is despite bullying perpetration generally being under-reported due to social desirability bias (i.e., a bias against self-reporting aggressive behaviour towards peers) or cognitive distortion (i.e., a self-serving rationalising attitude or belief to neutralise, defend, or minimise actions) [32, 58, 59].

It is also possible that the impact of the COVID-19 pandemic on school attendance during 2021 may have lowered an adolescent’s potential risk of being bullied. School closures varied greatly between and within the three countries across the COVID-19 pandemic. Although most affected schools had mostly returned to normal by this time, it is still possible that adolescents may not have been physically attending school or attending on a more limited basis, therefore reducing their potential exposure. It is also possible that the upheaval to school attendance altered the social dynamics within peer groups. Further, for Vietnam specifically, the delay to data collection meant that the recall period (past three months) of the bullying questions mostly consisted of the summer school holidays where adolescents weren’t at school. However, if this was a driving factor, this pattern would also have been seen for bullying perpetration which did not appear to be the case. There also did not appear to be a corresponding uptick in cyberbullying victimisation or perpetration which may have been expected if there was less opportunity for face-to-face bullying. Without baseline estimates, it is difficult to determine the extent of any impact of COVID-19 on the estimates reported in this study.

Interestingly, available estimates from the most recent Vietnam GSHS 2019 showed a substantial decrease in bullying victimisation as compared to the Vietnam GSHS 2013, with prevalence dropping from 22.6 to 6.2% among 13–17-year-olds (noting these estimates employed a more liberal operationalisation of repetition) [60]. Among students, negative attitudes towards bullying perpetration and bystanding, and favourable attitudes towards bullying defending, are associated with reductions in bullying behaviours in schools [61, 62]. It is possible that the increased global awareness of the harm attributable to bullying is changing adolescent attitudes towards bullying, leading to reductions in prevalence. This may also provide some insight into the differences seen between the findings of NAMHS and equivalent yet older GSHS findings.

Both Kenya and Indonesia demonstrated a significant difference by age, whereby younger adolescents aged 10–14 years (Kenya: 4.9%; Indonesia: 3.4%) were more likely than older adolescents aged 15–17 years (Kenya: 2.6%; Indonesia: 1.1%) to have been victimised, albeit noting the comparison between a five-year and three-year age group. However, this decrease by age is consistent with what has been previously reported, i.e., where younger ages tend to report more bullying victimisation [63–65]. In contrast, no difference by age was seen for bullying perpetration although again this is also broadly consistent with the literature [63]. No difference was found by sex except for bullying perpetration in Vietnam. This in line with previous studies suggesting differences by sex are less apparent in adolescence as compared to childhood [16, 17]. Further, the profile of victimisation and perpetration was relatively consistent between countries, albeit with some exceptions, e.g., 22.2% of those who had been bullied reported physical victimisation in Indonesia as compared to 58.6% in Kenya and 55.5% in Vietnam. The lower proportions of adolescents endorsing cyber victimisation, as compared to other forms of victimisation, reflects similar patterns found in population-level prevalence estimates from other studies. For example, a study utilising HBSC data for 40 countries (inclusive of HICs and LMICs) reported pooled prevalence of 8.0% for traditional bullying only, 2.3% for cyberbullying victimisation only, and 1.7% for traditional and cyberbullying combined [23].

Broadly, the associations between bullying victimisation and mental disorders found in NAMHS (Kenya: aOR 2.2; Indonesia: aOR 3.6; Vietnam: aOR 4.7) were on par, if not stronger, than those available from other large-scale studies. For example, data from the Eurasian Child Mental Health Study showed significantly increased odds of externalising symptoms among females (OR 1.13) and males (OR 1.08) in Indonesia who experienced bullying victimisation as compared to those who had not, although only males who had been victimised demonstrated increased odds of internalising symptoms (OR 1.05) compared to those who had not [66]. While this may partly be a function of the relatively low prevalence of both bullying victimisation and mental disorders reported in NAMHS [39], intervention studies have previously reported a ‘healthy context paradox’, whereby the association between bullying victimisation and mental disorders is stronger within schools receiving a successful bullying intervention (i.e., where the prevalence of victimisation decreases), compared to schools not receiving the intervention [67, 68]. Even though the prevalence of bullying victimisation reported in NAMHS was comparatively low, it was still significantly associated with mental disorders, perhaps even more so if these findings are taken to indicate that bullying victimisation may be a less normative experience. Additionally, the association found between bullying perpetration and mental disorders in NAMHS (Kenya: aOR 4.4; Indonesia: aOR 4.2) was also significant, despite the limited available evidence suggesting that the impact of victimisation on mental health is greater than perpetration [69].

The findings presented in this study highlight the importance of considering both bullying victimisation and perpetration when considering bullying interventions as a means of addressing poor mental health among adolescents. Previous reviews suggest that complex, whole-of-school (involving school staff, students, and parents/caregivers) and multi-tiered (with universal prevention and targeted intervention elements) programs are effective in reducing bullying in HICs [70–72]. Equivalent evidence for bullying interventions within LMICs is extremely limited. A recent systematic review of studies evaluating the effectiveness of school-based bullying interventions only found three studies (from Romania, Malaysia, and South Africa, respectively), with results mixed and unable to provide an indication of overall effectiveness [73]. However, taking a holistic view of relevant studies can potentially provide a starting point for focusses or targets of bullying interventions in LMICs. For example, a systematic review of school-based bullying interventions within HICs found that specific components of interventions, such as information for parents and informal peer involvement, were associated with significant reductions in bullying [74]. In parallel, an analysis of GSHS data from 83 countries (inclusive of LMICs and HICs) found that higher levels of parental and peer support, respectively, were associated with a significantly reduced odds of bullying victimisation [26]. In combination, these studies point towards peer and parental support as a target or component of bullying interventions in LMICs. However, substantially more research from LMICs, including from the three countries involved in NAMHS which have little to no data for bullying interventions, is required to determine whether such interventions would be effective and how these could be operationalised across different cultural contexts.

Several limitations must be considered when interpreting the findings presented in this study. First, it is widely acknowledged that both bullying victimisation and perpetration have a bidirectional relationship with mental disorders i.e., where those with existing poor mental health may be more likely to be bullied or bullying others [21, 22, 75–78]. The ability to further investigate this in the context of NAMHS is limited by the cross-sectional nature of the study and must be considered when interpreting these findings. However, longitudinal studies, including studies from LMICs [79], have found strong associations between bullying and mental health after addressing this bidirectionality, further supporting the interpretation of the associations reported in NAMHS. Second, the small numbers of adolescents reporting victimisation-perpetration, particularly in Vietnam and Indonesia, meant that there was insufficient statistical power to assess the association between this subgroup and mental disorders. This is a notable limitation given previous studies have found that those reporting both bullying victimisation and perpetration have different outcomes as compared to those only reporting victimisation or perpetration [80–82]. A similar limitation exists regarding the ability to assess associations by mental disorder type (e.g., internalising vs. externalising disorders) as well as by demographic characteristics (e.g., age group). However, while the aim of the current study was to provide consistent cross-national profiles of bullying victimisation and perpetration, future analysis of NAMHS data can focus on the countries among the three sampled which have sufficient numbers to explore these questions. Finally, a disconnect exists between the overall or ‘global’ bullying question and the subsequent questions about the subtypes. For example, adolescents were able to endorse the global bullying question and then respond ‘Never’ to all subtypes. Similarly, consistency was not required between the frequency of bullying reported at the global level and the cumulative frequency of the combined subtypes. The choice of including both a global and specific subtype questions was made to ensure as much information about bullying was gathered during NAMHS, while acknowledging the potential for discrepancies [41]. The current study utilised data on the subtypes as a means of profiling the experience of those involved in bullying and comparing this across countries. Analysis of the prevalence of the different subtypes of bullying victimisation and perpetration among the population are planned for future publications.

## Conclusions

This study is the first to collectively report nationally representative prevalence estimates for bullying victimisation and perpetration among adolescents aged 10–17 years in Kenya, Indonesia, and Vietnam. The prevalence of bullying victimisation and perpetration differed between the three countries, yet the association with mental disorders remained largely consistent. The findings of this study speak to the importance of considering bullying victimisation and perpetration when looking to improve adolescent mental health at a population level, as well as the need for timely and country-specific data for LMICs which utilises high quality sampling and measurement to better understand bullying in these contexts.

## Supplementary Information

Supplementary Material 1

## Data Availability

The NAMHS datasets are co-owned by the University of Queensland (UQ) and each respective in-country lead organisation (K-NAMHS: African Population and Health Research Center [APHRC] and UQ; I-NAMHS: UGM and UQ; V-NAMHS: Institute of Sociology [IOS] and UQ). Currently, these datasets or analysis of these datasets are available for collaborative work on request to the relevant data owners following an established protocol. Work is currently underway to convert the NAMHS datasets into public use datasets, allowing for wide use of these datasets while ensuring protection of participant privacy, adherence to country-specific legislation, and appropriate use of data. This includes development of accompanying meta-data, inclusive of a codebook, technical manual, and analysis files. The expected launch date for these public use datasets and accompanying meta-data is 2026, with hosting mechanisms currently under development in line with country legislation and ethical requirements.

## Abbreviations

ADHD: Attention-deficit/hyperactivity disorder
aOR: Adjusted odds ratio
BCS-A: Bullying and Cyberbullying Scale for Adolescents
CIs: Confidence intervals
DHS: Demographic and Health Surveys
DISC-5: Diagnostic Interview Schedule for Children, Version 5
DSM-5: Diagnostic and Statistical Manual of Mental Disorders, Fifth Edition
GBD: Global Burden of Disease Study
GSHS: Global School-Based Student Health Survey
HBSC: Health Behaviour in School-aged Children
HICs: High-income countries
LMICs: Low- and middle-income countries
NAMHS: National Adolescent Mental Health Surveys
OR: Odds ratio
SDGs: Sustainable Development Goals

## Declarations

### About this supplement

This article has been published as part of Child and Adolescent Psychiatry and Mental Health, Volume 19 Supplement S1, 2025: Findings from the National Adolescent Mental Health Surveys: Factors influencing adolescent mental health in Kenya, Indonesia, and Vietnam. The full contents of the supplement are available at https://capmh.biomedcentral.com/articles/supplements/volume-19-supplement-1.

## References

[1] Olweus D. Bully/victim problems in school: facts and intervention. Eur J Psychol Educ. 1997;12(4):495–510.

[2] United Nations. 4 Ensure inclusive and equitable quality education and promote lifelong learning opportunities for all 2023. Available from: https://sdgs.un.org/goals/goal4

[3] Cornu C, Liu Y. TCG4: development of SDG thematic indicator 4.a.2. Dubai. United Arab Emirates: Section of Health and Education, Division for Inclusion, Peace and Sustainable Development, UNESCO; 2018.

[4] UNESCO Institute for Statistics. Percentage of students experiencing bullying in the last 12 months in a) primary and b) lower secondary education: UNESCO Institute for Statistics; 2023. Available from: https://uis.unesco.org/en/glossary-term/percentage-students-experiencing-bullying-last-12-months-primary-and-b-lower-secondary

[5] GBD 2019 Risk Factors Collaborators. Global burden of 87 risk factors in 204 countries and territories, 1990–2019: a systematic analysis for the global burden of disease study 2019. Lancet. 2020;396(10258):1223–49. 33069327 10.1016/S0140-6736(20)30752-2 PMC7566194

[6] Jadambaa A, Thomas HJ, Scott JG, Graves N, Brain D, Pacella R. The contribution of bullying victimisation to the burden of anxiety and depressive disorders in Australia. Epidemiol Psychiatr Sci. 2019;29:e54. 31533868 10.1017/S2045796019000489 PMC8061250

[7] Moore SE, Norman RE, Suetani S, Thomas HJ, Sly PD, Scott JG. Consequences of bullying victimization in childhood and adolescence: A systematic review and meta-analysis. World J Psychiatry. 2017;7(1):60–76. 28401049 10.5498/wjp.v7.i1.60 PMC5371173

[8] Kretschmer T, Veenstra R, Deković M, Oldehinkel AJ. Bullying development across adolescence, its antecedents, outcomes, and gender-specific patterns. Dev Psychopathol. 2017;29(3):941–55. 27417540 10.1017/S0954579416000596

[9] Gibb SJ, Horwood LJ, Fergusson DM. Bullying victimization/perpetration in childhood and later adjustment: findings from a 30 year longitudinal study. J Aggress Confl Peace Res. 2011;3(2):82–8.

[10] Hemphill SA, Kotevski A, Herrenkohl TI, Bond L, Kim MJ, Toumbourou JW, et al. Longitudinal consequences of adolescent bullying perpetration and victimisation: A study of students in Victoria, Australia. Criminal Behav Mental Health. 2011;21(2):107–16. 10.1002/cbm.802 PMC3760487 21370296

[11] Copeland WE, Wolke D, Angold A, Costello EJ. Adult psychiatric outcomes of bullying and being bullied by peers in childhood and adolescence. JAMA Psychiatry. 2013;70(4):419–26. 23426798 10.1001/jamapsychiatry.2013.504 PMC3618584

[12] Sigurdson JF, Undheim AM, Wallander JL, Lydersen S, Sund AM. The long-term effects of being bullied or a bully in adolescence on externalizing and internalizing mental health problems in adulthood. Child Adolesc Psychiatry Ment Health. 2015;9:42. 26300969 10.1186/s13034-015-0075-2 PMC4546259

[13] Takizawa R, Maughan B, Arseneault L. Adult health outcomes of childhood bullying victimization: evidence from a five-decade longitudinal British birth cohort. Am J Psychiatry. 2014;171(7):777–84. 24743774 10.1176/appi.ajp.2014.13101401

[14] Thomas HJ, Chan GC, Scott JG, Connor JP, Kelly AB, Williams J. Association of different forms of bullying victimisation with adolescents’ psychological distress and reduced emotional wellbeing. Aust N Z J Psychiatry. 2016;50(4):371–9. 26296367 10.1177/0004867415600076

[15] Turner MG, Exum ML, Brame R, Holt TJ. Bullying victimization and adolescent mental health: general and typological effects across sex. J Criminal Justice. 2013;41(1):53–9.

[16] Cosma A, Bjereld Y, Elgar FJ, Richardson C, Bilz L, Craig W, et al. Gender differences in bullying reflect societal gender inequality: A multilevel study with adolescents in 46 countries. J Adolesc Health. 2022;71(5):601–8. 35817675 10.1016/j.jadohealth.2022.05.015

[17] Smith PK, López-Castro L, Robinson S, Görzig A. Consistency of gender differences in bullying in cross-cultural surveys. Aggress Violent Beh. 2019;45:33–40.

[18] Hellström L, Beckman L. Adolescents’ perception of gender differences in bullying. Scand J Psychol. 2020;61(1):90–6. 30690741 10.1111/sjop.12523 PMC7003756

[19] Loch AP, Astolfi RC, Leite MA, Papa CHG, Ryngelblum M, Eisner M, et al. Victims, bullies and bully–victims: prevalence and association with negative health outcomes from a cross-sectional study in São Paulo, Brazil. Int J Public Health. 2020;65(8):1485–95. 33025092 10.1007/s00038-020-01481-5

[20] Moore SE, Norman RE, Sly PD, Whitehouse AJ, Zubrick SR, Scott J. Adolescent peer aggression and its association with mental health and substance use in an Australian cohort. J Adolesc. 2014;37(1):11–21. 24331300 10.1016/j.adolescence.2013.10.006

[21] Azevedo Da Silva M, Gonzalez JC, Person GL, Martins SS. Bidirectional association between bullying perpetration and internalizing problems among youth. J Adolesc Health. 2020;66(3):315–22. 31780386 10.1016/j.jadohealth.2019.09.022 PMC7285807

[22] He Y, Chen S-S, Xie G-D, Chen L-R, Zhang T-T, Yuan M-Y, et al. Bidirectional associations among school bullying, depressive symptoms and sleep problems in adolescents: A cross-lagged longitudinal approach. J Affect Disord. 2022;298:590–8. 34800574 10.1016/j.jad.2021.11.038

[23] Biswas T, Thomas HJ, Scott JG, Munir K, Baxter J, Huda MM, et al. Variation in the prevalence of different forms of bullying victimisation among adolescents and their associations with family, peer and school connectedness: a population-based study in 40 lower and middle income to high-income countries (LMIC-HICs). J Child Adolesc Trauma. 2022;15(4):1029–39. 36439674 10.1007/s40653-022-00451-8 PMC9684371

[24] Zych I, Farrington DP, Llorent VJ, Ttofi MM. School bullying in different countries: prevalence, risk factors, and Short-Term outcomes. In: Zych I, Farrington DP, Llorent VJ, Ttofi MM, editors. Protecting children against bullying and its consequences. Cham: Springer International Publishing; 2017. pp. 5–22.

[25] Nguyen AJ, Bradshaw C, Townsend L, Bass J. Prevalence and correlates of bullying victimization in four Low-Resource countries. J Interpers Violence. 2020;35(19–20):3767–90. 29294770 10.1177/0886260517709799

[26] Biswas T, Scott JG, Munir K, Thomas HJ, Huda MM, Hasan MM, et al. Global variation in the prevalence of bullying victimisation amongst adolescents: role of peer and parental supports. EClinicalMedicine. 2020;20:100276. 32300737 10.1016/j.eclinm.2020.100276 PMC7152826

[27] Craig W, Harel-Fisch Y, Fogel-Grinvald H, Dostaler S, Hetland J, Simons-Morton B, et al. A cross-national profile of bullying and victimization among adolescents in 40 countries. Int J Public Health. 2009;54(2):216–24. 19623475 10.1007/s00038-009-5413-9 PMC2747624

[28] Pernille, Due. Bjørn Evald Holstein. Bullying victimization among 13 to 15 year old school children: results from two comparative studies in 66 countries and regions. Int J Adolesc Med Health. 2008;20(2):209–22. 10.1515/ijamh.2008.20.2.209 18714557

[29] World Health Organisation. Global school-based student health survey (GSHS) Geneva, Switzerland: World Health Organisation. 2023. Available from: https://www.who.int/teams/noncommunicable-diseases/surveillance/systems-tools/global-school-based-student-health-survey

[30] Bischops AC, Radev ST, Köthe U, Chen S, Geldsetzer P, Sarker M, et al. Data resource profile: the global School-based student health Survey—behavioural risk and protective factors among adolescents. Int J Epidemiol. 2022;52(2):e102–9. 10.1093/ije/dyac208 36350584

[31] Roberts C, Freeman J, Samdal O, Schnohr CW, de Looze ME, Nic Gabhainn S, et al. The health behaviour in School-aged children (HBSC) study: methodological developments and current tensions. Int J Public Health. 2009;54(Suppl 2):140–50. 19639259 10.1007/s00038-009-5405-9 PMC2732766

[32] Thomas HJ, Connor JP, Lawrence DM, Hafekost JM, Zubrick SR, Scott JG. Prevalence and correlates of bullying victimisation and perpetration in a nationally representative sample of Australian youth. Australian New Z J Psychiatry. 2017;51(9):909–20. 28513190 10.1177/0004867417707819

[33] Olweus D. School bullying: development and some important challenges. Annu Rev Clin Psychol. 2013;9:751–80. 23297789 10.1146/annurev-clinpsy-050212-185516

[34] Thomas HJ, Connor JP, Scott JG. Integrating traditional bullying and cyberbullying: challenges of definition and measurement in Adolescents– a review. Educational Psychol Rev. 2015;27(1):135–52.

[35] Vivolo-Kantor AM, Martell BN, Holland KM, Westby R. A systematic review and content analysis of bullying and cyber-bullying measurement strategies. Aggress Violent Behav. 2014;19(4):423–34. 26752229 10.1016/j.avb.2014.06.008 PMC4703330

[36] Olweus D. The Revised Olweus Bully/Victim Questionnaire. Bergen: University of Bergen, Research Center for Health Promotion. 1996.

[37] Solberg ME, Olweus D. Prevalence Estimation of school bullying with the Olweus bully/victim questionnaire. Aggressive Behav. 2003;29(3):239–68.

[38] Scott JG, Moore SE, Sly PD, Norman RE. Bullying in children and adolescents: A modifiable risk factor for mental illness. Australian New Z J Psychiatry. 2014;48(3):209–12. 24317152 10.1177/0004867413508456

[39] Erskine HE, Maravilla JC, Wado YD, Wahdi AE, Loi VM, Fine SL, et al. Prevalence of adolescent mental disorders in Kenya, Indonesia, and Viet Nam measured by the National adolescent mental health surveys (NAMHS): a multi-national cross-sectional study. Lancet. 2024;403(10437):1671–80. 38588689 10.1016/S0140-6736(23)02641-7

[40] Erskine HE, Blondell SJ, Enright ME, Shadid J, Wado YD, Wekesah FM, et al. Measuring the prevalence of mental disorders in adolescents in Kenya, Indonesia, and Vietnam: study protocol for the National adolescent mental health surveys. J Adolesc Health. 2023;72(1, Supplement):S71–8. 36229399 10.1016/j.jadohealth.2021.05.012

[41] Thomas HJ, Scott JG, Coates JM, Connor JP. Development and validation of the bullying and cyberbullying scale for adolescents: A multi-dimensional measurement model. Br J Educ Psychol. 2019;89(1):75–94. 29726005 10.1111/bjep.12223

[42] Hunter SC, Boyle JME, Warden D. Perceptions and correlates of peer-victimization and bullying. Br J Educ Psychol. 2007;77(4):797–810. 17971286 10.1348/000709906X171046

[43] Smith PK. Bullying in schools: Thirty years of research. Bullying in different contexts. New York, NY, US: Cambridge University Press; 2011. pp. 36–60.

[44] Bitsko R, Adams HR, Holbrook J, Jones C, Augustine E, Mink J, et al. 2.50 DIAGNOSTIC INTERVIEW SCHEDULE FOR CHILDREN, VERSION 5 (DISC-5): DEVELOPMENT AND VALIDATION OF ADHD AND TIC DISORDER MODULES. J Am Acad Child Adolesc Psychiatry. 2019;58(10):S187.

[45] Shaffer D, Fisher P, Lucas CP, Dulcan MK, Schwab-Stone ME. NIMH diagnostic interview schedule for children version IV (NIMH DISC-IV): description, differences from previous versions, and reliability of some common diagnoses. J Am Acad Child Adolesc Psychiatry. 2000;39(1):28–38. 10638065 10.1097/00004583-200001000-00014

[46] American Psychiatric Association. Diagnostic and Statistical Manual of Mental Disorders, Fifth Edition. Arlington, VA: American Psychiatric Association; 2013.

[47] The Demographic and Health Surveys Program. Wealth Index Construction Maryland, USA: The Demographic and Health Surveys Program. 2022. Available from: https://dhsprogram.com/topics/wealth-index/Wealth-Index-Construction.cfm

[48] GBD 2019 Mental Disorders Collaborators. Global, regional, and National burden of 12 mental disorders in 204 countries and territories, 1990–2019: a systematic analysis for the global burden of disease study 2019. Lancet Psychiatry. 2022;9(2):137–50. 35026139 10.1016/S2215-0366(21)00395-3 PMC8776563

[49] Fine SL, Musci RJ, Bass JK, Chipeta E, Mafuta EM, Pinandari AW, et al. A Multi-Country study of risk and protective factors for emotional and behavioral problems among early adolescents. J Adolesc Health. 2022;71(4):480–7. 35710891 10.1016/j.jadohealth.2022.05.002 PMC9477503

[50] StataCorp. Stata statistical software: release 17. College station. TX: StataCorp LLC; 2021.

[51] World Health Organisation, Ministry of Health. Kenya 2003 Global School-based Student Health Survey Geneva, Switzerland: World Health Organisation. 2019 [updated 03/05/2019. Available from: https://extranet.who.int/ncdsmicrodata/index.php/catalog/13/data-dictionary/F5?file_name=KEH2003_public_use

[52] World Health Organization, Health Research and Development Agency, Prevention UCfDCa. Indonesia 2015 Global School-based Student Health Survey Geneva, Switzerland: World Health Organisation,; 2019 [updated 13/06/2019. Available from: https://extranet.who.int/ncdsmicrodata/index.php/catalog/489/data-dictionary/F2?file_name=IDN2015_Public_Use_national

[53] World Health Organisation, Hanoi Medical University. Vietnam 2013 Global School-based Student Health Survey Geneva, Switzerland: World Health Organisation. 2019. updated 13/06/2019. Available from: https://extranet.who.int/ncdsmicrodata/index.php/catalog/482/data-dictionary/F1?file_name=VNM2013_Public_Use

[54] Kelly CA, Soler-Hampejsek E, Mensch BS, Hewett PC. Social desirability bias in sexual behavior reporting: evidence from an interview mode experiment in rural Malawi. Int Perspect Sex Reprod Health. 2013;39(1):14–21. 23584464 10.1363/3901413 PMC4023461

[55] Le LC, Blum RW, Magnani R, Hewett PC, Do HM. A pilot of audio computer-assisted self-interview for youth reproductive health research in Vietnam. J Adolesc Health. 2006;38(6):740–7. 16730604 10.1016/j.jadohealth.2005.07.008

[56] Lebrun-Harris LA, Sherman LJ, Limber SP, Miller BD, Edgerton EA. Bullying victimization and perpetration among U.S. Children and adolescents: 2016 National survey of children’s health. J Child Fam Stud. 2019;28(9):2543–57.

[57] Shahrour G, Dardas LA, Al-Khayat A, Al-Qasem A. Prevalence, correlates, and experiences of school bullying among adolescents: A National study in Jordan. School Psychol Int. 2020;41(5):430–53.

[58] Cornell DG, Brockenbrough K. Identification of bullies and victims. J School Violence. 2004;3(2–3):63–87.

[59] Finkelhor D, Shattuck A, Turner H, Hamby S. A behaviorally specific, empirical alternative to bullying: aggravated peer victimization. J Adolesc Health. 2016;59(5):496–501. 27444868 10.1016/j.jadohealth.2016.05.021

[60] World Health Organisation Western Pacific Region. Vietnam ministry of health, training VMoEa. Report of the 2019 global School-based student health survey in Viet Nam. Vietnam: Hanoi; 2022.

[61] López DP, López-Nicolás R, López-López R, Puente-López E, Ruiz-Hernández JA. Association between attitudes toward violence and violent behavior in the school context: A systematic review and correlational meta-analysis. Int J Clin Health Psychol. 2022;22(1):100278. 34934422 10.1016/j.ijchp.2021.100278 PMC8640117

[62] Man X, Liu J, Xue Z. Does bullying attitude matter in school bullying among adolescent students: evidence from 34 OECD countries. Children (Basel). 2022;9(7):975. 10.3390/children9070975 PMC9319614 35883961

[63] Armitage R. Bullying in children: impact on child health. BMJ Paediatr Open. 2021;5(1):e000939. 10.1136/bmjpo-2020-000939 PMC7957129 33782656

[64] Australian Institute of. Health and welfare. Australia’s children. Canberra, Australia: AIHW; 2020.

[65] UNESCO. Behind the numbers: ending school violence and bullying. Paris, France: United Nations Education, Scientific, and Cultural Organization; 2019.

[66] Chudal R, Tiiri E, Brunstein Klomek A, Ong SH, Fossum S, Kaneko H, et al. Victimization by traditional bullying and cyberbullying and the combination of these among adolescents in 13 European and Asian countries. Eur Child Adolesc Psychiatry. 2022;31(9):1391–404. 33884501 10.1007/s00787-021-01779-6 PMC9402766

[67] Huitsing G, Lodder GMA, Oldenburg B, Schacter HL, Salmivalli C, Juvonen J, et al. The healthy context paradox: victims’ adjustment during an Anti-Bullying intervention. J Child Fam Stud. 2019;28(9):2499–509.

[68] Salmivalli C, Laninga-Wijnen L, Malamut ST, Garandeau CF. Bullying prevention in adolescence: solutions and new challenges from the past decade. J Res Adolescence. 2021;31(4):1023–46. 10.1111/jora.12688 PMC9271952 34820956

[69] McKay MT, Cannon M, Chambers D, Conroy RM, Coughlan H, Dodd P, et al. Childhood trauma and adult mental disorder: A systematic review and meta-analysis of longitudinal cohort studies. Acta Psychiatrica Scandinavica. 2021;143(3):189–205. 33315268 10.1111/acps.13268

[70] Gaffney H, Ttofi MM, Farrington DP. Evaluating the effectiveness of school-bullying prevention programs: an updated meta-analytical review. Aggress Violent Beh. 2019;45:111–33.

[71] Ttofi MM, Farrington DP. Effectiveness of school-based programs to reduce bullying: a systematic and meta-analytic review. J Experimental Criminol. 2011;7(1):27–56.

[72] Vreeman RC, Carroll AE. A systematic review of school-based interventions to prevent bullying. Arch Pediatr Adolesc Med. 2007;161(1):78–88. 17199071 10.1001/archpedi.161.1.78

[73] Sivaraman B, Nye E, Bowes L. School-based anti-bullying interventions for adolescents in low- and middle-income countries: A systematic review. Aggress Violent Beh. 2019;45:154–62.

[74] Gaffney H, Ttofi MM, Farrington DP. What works in anti-bullying programs? Analysis of effective intervention components. J Sch Psychol. 2021;85:37–56. 33715780 10.1016/j.jsp.2020.12.002

[75] Hamstra C, Fitzgerald M. Longitudinal effects from childhood abuse to bullying perpetration in adolescence: the role of mental health and social problems. J Child Adolesc Trauma. 2022;15(3):869–81. 35958700 10.1007/s40653-021-00409-2 PMC9360357

[76] Brunstein Klomek A, Barzilay S, Apter A, Carli V, Hoven CW, Sarchiapone M, et al. Bi-directional longitudinal associations between different types of bullying victimization, suicide ideation/attempts, and depression among a large sample of European adolescents. J Child Psychol Psychiatry. 2019;60(2):209–15. 30024024 10.1111/jcpp.12951

[77] Kljakovic M, Hunt C. A meta-analysis of predictors of bullying and victimisation in adolescence. J Adolesc. 2016;49:134–45. 27060847 10.1016/j.adolescence.2016.03.002

[78] Reijntjes A, Kamphuis JH, Prinzie P, Telch MJ. Peer victimization and internalizing problems in children: A meta-analysis of longitudinal studies. Child Abuse Negl. 2010;34(4):244–52. 20304490 10.1016/j.chiabu.2009.07.009

[79] Le HTH, Tran N, Campbell MA, Gatton ML, Nguyen HT, Dunne MP. Mental health problems both precede and follow bullying among adolescents and the effects differ by gender: a cross-lagged panel analysis of school-based longitudinal data in Vietnam. Int J Mental Health Syst. 2019;13(1):35. 10.1186/s13033-019-0291-x PMC6525446 31131020

[80] Özdemir M, Stattin H. Bullies, victims, and bully-victims: a longitudinal examination of the effects of bullying‐victimization experiences on youth well‐being. J Aggress Confl Peace Res. 2011;3(2):97–102.

[81] Holt MK, Vivolo-Kantor AM, Polanin JR, Holland KM, DeGue S, Matjasko JL, et al. Bullying and suicidal ideation and behaviors: A Meta-Analysis. Pediatrics. 2015;135(2):e496–509. 25560447 10.1542/peds.2014-1864 PMC4702491

[82] O’Brennan LM, Bradshaw CP, Sawyer AL. Examining developmental differences in the social-emotional problems among frequent bullies, victims, and bully/victims. Psychol Sch. 2009;46(2):100–15.

