# Supplementary material for: Bullying victimisation and perpetration and the association with mental disorders among adolescents in Kenya, Indonesia, and Vietnam: Findings from the National Adolescent Mental Health Surveys
Source: Child Adolesc Psychiatry Ment Health. 2025 Jul 31;19(Suppl 1):87. doi: 10.1186/s13034-025-00922-4 (PMC12312254; doi:10.1186/s13034-025-00922-4)
Supplement: Supplementary file 1 — Supplementary Material 1 [file 13034_2025_922_MOESM1_ESM.docx]

**Additional File 1**

Table S1. Proportions and unweighted numbers of adolescents endorsing each response option for victimisation and perpetration in Kenya, Indonesia, and Vietnam.

|  | **Kenya** | | **Indonesia** | | **Vietnam** | |
| --- | --- | --- | --- | --- | --- | --- |
|  | **% (95% CI)** | **n** | **% (95% CI)** | **n** | **% (95% CI)** | **n** |
| **Victimisation** |  |  |  |  |  |  |
| Never | 88.7 (87.4 - 89.9) | 4,577 | 84.6 (81.4 - 87.3) | 4,911 | 92.6 (91.2 - 93.8) | 5,523 |
| Once or twice | 7.1 (6.3 - 8.0) | 358 | 11.8 (9.5 - 14.6) | 579 | 4.1 (3.3 - 5.1) | 286 |
| Every few weeks | 1.0 (0.7 - 1.4) | 51 | 0.9 (0.5 - 1.5) | 34 | 0.8 (0.5 - 1.2) | 49 |
| About once a week | 0.8 (0.6 - 1.3) | 45 | 0.3 (0.2 - 0.6) | 17 | 0.2 (0.1 - 0.4) | 10 |
| A few days a week | 1.1 (0.8 - 1.5) | 61 | 0.8 (0.5 - 1.4) | 41 | 0.1 (0.1 - 0.3) | 10 |
| Most days | 1.2 (0.9 - 1.6) | 60 | 0.5 (0.3 - 0.9) | 28 | 0.1 (<0.1 - 0.4) | 5 |
| Don’t know | np | 1 | 0.9 (0.5 - 1.6) | 48 | 1.6 (1.2 - 2.0) | 87 |
| Prefer not to say | np | 2 | 0.2 (0.1 - 0.5) | 6 | 0.6 (0.4 - 0.9) | 26 |
| **Perpetration** |  |  |  |  |  |  |
| Never | 93.2 (92.2 - 94.1) | 4,784 | 90.1 (87 - 92.6) | 5,202 | 94.1 (92.7 - 95.2) | 5,611 |
| Once or twice | 3.6 (3.0 - 4.2) | 197 | 7.9 (5.8 - 10.6) | 356 | 4 (3.1 - 5.1) | 258 |
| Every few weeks | 0.7 (0.5 - 1.1) | 42 | 0.6 (0.3 - 1.2) | 26 | 0.5 (0.3 - 1.0) | 38 |
| About once a week | 0.5 (0.3 - 0.8) | 30 | 0.2 (0.1 - 0.4) | 9 | 0.1 (<0.1 - 0.2) | 6 |
| A few days a week | 1.0 (0.7 - 1.3) | 53 | 0.2 (0.1 - 0.6) | 19 | 0.1 (<0.1 - 0.2) | 7 |
| Most days | 0.9 (0.6 - 1.2) | 43 | 0.2 (<0.1 - 0.5) | 12 | 0.1 (<0.1 - 0.3) | 5 |
| Don’t know | 0.1 (<0.1 - 0.2) | 5 | 0.8 (0.4 - 1.7) | 36 | 0.8 (0.5 - 1.2) | 50 |
| Prefer not to say | np | 1 | np | 4 | 0.4 (0.2 - 0.6) | 21 |

Proportions (%) and 95% CIs are weighted. Numbers (n) are unweighted. Proportions are listed as ‘not published’ (np) where n < 5.
